# Hypersensitive C‐reactive protein‐albumin ratio is associated with stroke‐associated pneumonia and early clinical outcomes in patients with acute ischemic stroke

**DOI:** 10.1002/brb3.2675

**Published:** 2022-06-24

**Authors:** Lingling Huang, Rong Zhang, Jiahui Ji, Fengdan Long, Yadong Wang, Juan Lu, Ge Xu, Yaming Sun

**Affiliations:** ^1^ Department of Neurology Zhangjiagang TCM Hospital Affiliated to Nanjing University of Chinese Medicine Zhangjiagang China

**Keywords:** A^2^DS^2^ score, acute ischemic stroke, hypersensitive C‐reactive protein‐albumin ratio, stroke‐associated pneumonia

## Abstract

**Objectives:**

We aimed to explore the association between the baseline hypersensitive C‐reactive protein‐albumin ratio (CAR) and stroke‐associated pneumonia (SAP) during hospitalization and the short‐term prognosis in patients with acute ischemic stroke (AIS).

**Methods:**

We enrolled 766 patients with AIS and collected their admission baseline characteristics, including their National Institutes of Health Stroke Scale score, CAR, age, atrial fibrillation, dysphagia, sex, stroke severity (A^2^DS^2^) score, and other information. The occurrence of SAP within 7 days after stroke, length of hospital stay, and physical condition at discharge were also recorded. The patients’ Modified Rankin Scale (mRS) scores and mortality 3 months after AIS were further evaluated at follow‐up. All patients were divided into four groups based on the quartiles of the admission CAR (Q1 <1.3, Q2 1.3–3.7, Q3 3.7–9.3, Q4 ≥9.3).

**Results:**

During hospitalization, 92 (11.9%) patients were diagnosed with SAP. The patients with SAP had a higher CAR than the non‐SAP patients (*p* < .001). In the multivariate‐adjusted model, the patients in the Q3 and Q4 groups had a higher SAP risk (aOR was 5.21 and 17.72, *p*‐trend < .001) than those in the lowest quartile. The area under the curve for the CAR's ability to predict SAP was 0.810 in the receiver operating characteristic curve analysis and had a similar predictive efficacy as the A^2^DS^2^ score (*p <*.05). The length of stay in the SAP group was almost the same as that in the non‐SAP group, but the clinical outcomes were worse at discharge and at the 3‐month follow‐up in the SAP group. In addition, the patients in the higher CAR quartiles at admission were more likely to have poorer clinical outcomes.

**Conclusions:**

Patients with AIS with a high CAR at admission are more likely to develop SAP during hospitalization and have poor short‐term clinical outcomes. These findings might help to timely identify patients at high risk of SAP and provide a basis for further research on prophylactic antibiotic therapy.

## 1 INTRODUCTION

Stroke is the leading cause of acquired long‐term disability in adults and the second leading cause of death worldwide, resulting in a high social and family burden (Katan & Luft, 2018). In China, a prospective nationwide hospital‐based cohort study showed that the in‐hospital death rate was 1.9% (95% confidence interval [CI]: 1.7%−2.0%) for stroke inpatients, and the 12‐month fatality rate was 8.6% (95% CI: 8.3%−8.9%) for discharged stroke patients. The 12‐month disability rate was 16.6% (95% CI: 16.2%−17.0%) for stroke survivors, and the stroke recurrence rate was 5.7% (5.5%−6.0%) for stroke survivors (Tu et al., 2021). Stroke‐associated pneumonia (SAP) is a major risk factor for poor clinical outcomes and high mortality after stroke (Patel et al., 2020). Approximately 7%–38% of stroke patients in hospitals suffer from SAP, with the highest incidence occurring between 2 and 7 days (Nam et al., 2018). It causes prolonged hospitalization, delayed recovery, difficulty in executing rehabilitative procedures, poorer functional outcomes, higher mortality rates, and increased financial and care burdens on patients’ families (Hannawi et al., 2013; Katzan et al., 2007). In clinical practice, the diagnosis of SAP is often delayed for various reasons, which may lead to delayed or inappropriate antibiotic therapy (Esayag et al., 2010). Several studies have shown that there is no benefit in the preventive use of antibiotics (Kalra et al., 2015). Therefore, early risk assessment, SAP recognition, and anti‐infection treatment are recommended by the current guidelines to improve clinical outcomes (Smith et al., 2015).

To date, various SAP risk factors, such as sex, age, stroke severity, dysphagia, ventilator use, atrial fibrillation (AF), and diabetes mellitus, have been reported (Hannawi et al., 2013; Hotter et al., 2021; Patel et al., 2020). Several clinical prediction models, such as the age, atrial fibrillation, dysphagia, sex, stroke severity (A^2^DS^2^ (Tu et al., 2021)) score, Preventive ANtibacterial THERapy in acute Ischemic Stroke (PANTHERIS) score, and acute ischemic stroke‐associated pneumonia score (AIS‐APS) score, have been developed for early SAP prediction (Hotter et al., 2021). Among them, the A^2^DS^2^ score is commonly used in clinical practice because it is simple and effective (Helmy et al., 2016). Most SAP prediction models are based on clinical manifestations, with many scoring items, and some patients have atypical clinical manifestations, which influence the accuracy of the scoring methods and complicate SAP prediction. Therefore, a more facile and accurate SAP predictor is needed.

Previous studies have shown that the inflammatory response promotes SAP development (Hoffmann et al., 2017). In these studies, inflammatory biomarkers, such as the neutrophil‐to‐lymphocyte ratio (NLR) and monocyte‐to‐lymphocyte ratio (MLR), were explored to predict SAP (Cheng et al., 2020; Nam et al., 2018). C‐reactive protein (CRP), interleukin‐6 (IL‐6), and procalcitonin (PCT) have also been shown to be associated with SAP and can be used as predictors of SAP (Kwan et al., 2013; Xie et al., 2015). The hypersensitive C‐reactive protein‐albumin ratio (CAR) is calculated from hypersensitive C‐reactive protein (Hs‐CRP), and albumin (ALB) has been reported as a new and sensitive marker and is associated with poor outcomes in patients with tumors or cancer, critically ill patients, and patients with various brain diseases, such as spontaneous intracerebral hemorrhage (Bender et al., 2020; Park et al., 2018).

Our study aimed to investigate the relationship between the baseline CAR and SAP during hospitalization and short‐term prognosis in patients with AIS.

## 2 MATERIALS AND METHODS

### 2.1 Patients

**FIGURE 1:**
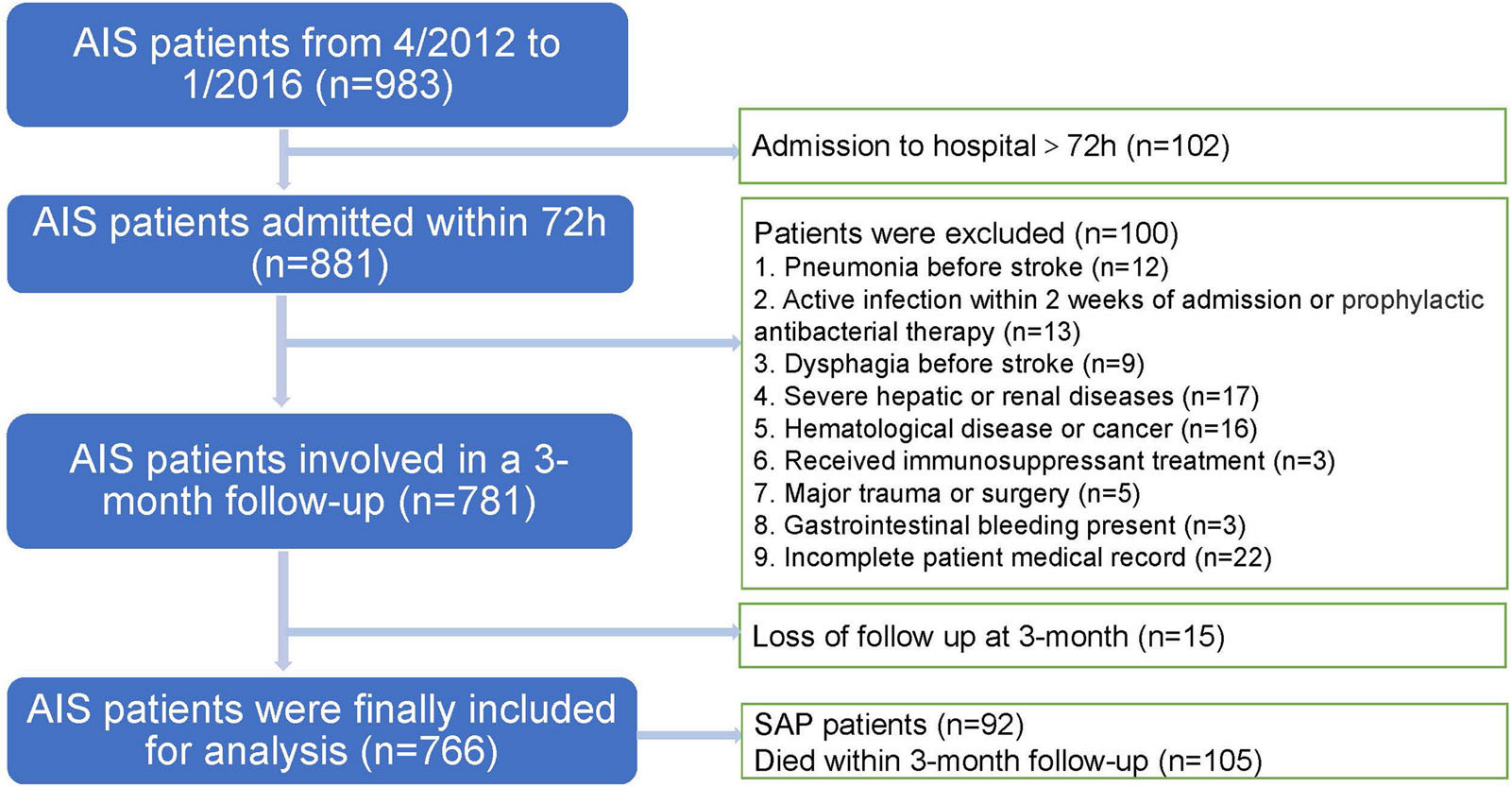
Research flowchart

We enrolled all patients with AIS who completed CRP and ALB tests on the day or within 24 ho after admission and who were treated in the emergency ward and neurology ward of the Zhangjiagang TCM Hospital affiliated to Nanjing University of Chinese Medicine from April 2012 to January 2016. All patients included in the study had stable vital signs on admission and no serious dysfunction of other organs (*n* = 983). Patients with AIS with unstable vital signs were not included in this study because they were admitted to the intensive care unit (ICU). AIS was diagnosed by doctors based on the patient's history, clinical features, and findings on computed tomography (CT) or magnetic resonance imaging (MRI) of the brain according to the criteria defined by the World Health Organization. Patients with acute ischemic stroke who were admitted within 72 h of the onset of symptoms were then selected for the research (*n* = 881). As the CAR is significantly affected by severe systemic inflammation, the additional exclusion criteria were as follows: (1) pneumonia before stroke (*n* = 12); (2) active infection within 2 weeks of admission or prophylactic antibacterial therapy (*n* = 13); (3) dysphagia before stroke (*n* = 9); (4) severe hepatic or renal diseases (*n* = 17); (5) hematological disease or cancer (*n* = 16); (6) received immunosuppressant treatment (*n* = 3); (7) major trauma or surgery (*n* = 5); (8) gastrointestinal bleeding present (*n* = 3); and (9) incomplete patient medical record (*n* = 22). Finally, 15 of the participants were lost to follow‐up at 3 months. Finally, we enrolled 766 patients with AIS in this study; 92 of them were diagnosed with SAP during hospitalization, and 105 died within 3 months of follow‐up, including six patients who died at the time of discharge (Figure 1).

### 2.2 Statement of ethics

We obtained approval from the Ethics Committee of the Zhangjiagang TCM Hospital Affiliated to Nanjing University of Chinese Medicine in China (No. 2020‐77‐1). Since our study was a retrospective data analysis and we obtained all the data from the clinical records without any clinical intervention, we did not obtain informed written consent from the patients.

### 2.3 Data collection

We collected the baseline information, including the patients’ demographics, such as sex and age. Known risk factors for cerebrovascular disease, such as history of stroke, hypertension, diabetes, atrial fibrillation, coronary heart disease, and smoking or alcohol consumption, were collected. The clinical data that were collected included the stroke subtype (TOAST classification), stroke severity (National Institutes of Health Stroke Scale, NIHSS), functional states (Modified Rankin Scale score, mRS) and A^2^DS^2^ score at admission. The thrombolytic therapy, medication use, imaging data, and other diagnosis‐related information were also recorded. The laboratory data of all participants were obtained after collecting venous blood when the patients were in the emergency department or in the ward, and the blood samples were collected within 24 h after hospital admission. The laboratory staff used the XE‐5000 (Mindray, China) to perform routine blood tests. The Hs‐CRP concentrations were detected using 5180CRP (Mindray). ALB and other biochemical indicators were tested by using an Olympus AU5400 automatic biochemical analyzer (First Chemical Co., Ltd., Japan).

### 2.4 Study outcomes

The primary outcome of our study was SAP. During hospitalization, patients who presented with symptoms or signs of a respiratory infection received routine blood tests and a chest CT scan to determine whether they had pneumonia. Doctors from the neurology and radiology department worked together to make the diagnosis of SAP, which was mainly based on the 2015 diagnostic criteria from the PISCES group (Smith et al., 2015). We also collected the total hospitalization days, NIHSS score, and mRS score at discharge, mRS score 3 months after discharge and 3‐month all‐cause mortality for all patients. Finally, we followed up with the patients themselves and their family members 3 months after discharge using telephone follow‐up to obtain their functional recovery (mRS score) and to determine whether they had died.

### 2.5 Statistical analysis

As mentioned above, we divided the patients into SAP and non‐SAP groups according to whether SAP occurred during their stay in the hospital. Patients were also divided into four groups based on the quartiles of admission CAR level (Q1 <1.3, Q2 1.3–3.7, Q3 3.7–9.3, Q4 ≥9.3). Finally, according to the results of the 3‐month follow‐up, they were divided into poor clinical outcomes (mRS 3–6) and good clinical outcomes (mRS 0–2), and they were also divided into two groups: those who died (mRS = 6) or those who did not die within 3 months (mRS 0–5).

We used SPSS (version 23.0; IBM, Armonk, NY, USA), MedCalc (version 13.0; MedCalc Software Ltd., Ostend, Belgium), and GraphPad Prism (version 8.0; GraphPad Software Inc., San Diego, CA, USA) to perform statistical analysis. Statistical significance was assumed when two‐tailed *p*‐values < .05. We used the Kolmogorov–Smirnov test to test whether the distribution of quantitative data was normal because the sample size was over 200. The mean ± standard deviation was used to express the normally distributed data, and Student's *t* test was performed to compare the intergroup differences that were statistically significant. The median (M) and first quartiles (Q_25_) and third quartiles (Q_75_) were used to express non‐normally distributed data, and the Mann–Whitney *U* test was used to compare the intergroup differences. Qualitative data are presented as percentages or ratios (%), and categorical variables were compared by the *χ* ^2^ test and Fisher's exact test for intergroup differences. Associations between the CAR quartiles and risk of SAP, 3‐month poor outcomes, and 3‐month all‐cause mortality were all performed by univariate and multivariate logistic regression analyses. In the multivariate logistic regression model, variables with *p* < .05 in the univariate analysis were adjusted to explore the independent effect of CAR on SAP. Taking the lowest quartile of CAR (Q1) as a reference, the odds ratios (ORs) and 95% CIs were calculated for each group. We used receiver operating characteristic (ROC) curves to evaluate the predictive value of the CAR and other biomarkers for SAP. A *Z*‐test was used to compare the areas under the ROC curves of the CAR and other biomarker values.

## 3 RESULTS

### 3.1 Comparison of the baseline data between the SAP and non‐SAP groups

The baseline demographic characteristics, medical history, and clinical and laboratory data are shown in Table 1. The SAP patients were older, had a longer waiting time at home after onset, had higher NIHSS scores on admission, and had a higher thrombolytic therapy rate. Additionally, the two groups had different AIS types. Patients with large artery atherosclerosis and cardioembolism were more common in the SAP group. The patients in the non‐SAP group were more likely to have arteriolar occlusion. The SAP patients had a higher dysphagia rate and increased nasogastric tube use than the non‐SAP patients. SAP patients were more likely to have AF and less likely to smoke or consume alcohol. Moreover, the A^2^DS^2^ score was higher in the SAP patients due to age, AF, dysphagia, sex, and stroke severity (NIHSS score). We did not observe statistically significant differences in sex, baseline blood pressure at admission, previous stroke history, diabetes history, or coronary heart disease history between the two groups (*p* > .05).

**TABLE 1:** Comparison of the baseline data between the stroke‐associated pneumonia (SAP) and non‐SAP groups

	Total (*n* = 776)	non‐SAP (*n* = 674)	SAP (*n* = 92)	Z/*χ* ^2^	*p*
**Demographic characteristics**					
Age (years), median (Q_25_–Q_75_)	71 (62–78)	69 (60–77)	79 (73–83)	−7.568	<.001
Sex (male), *n* (%)	411 (53.7%)	369 (54.7%)	42 (45.7%)	2.693	.118
**Clinical data**					
First symptom to admission time (hours), median (Q_25_–Q_75_)	12 (3–24)	12 (4–33)	5 (2–24)	−3.913	<.001
NIHSS score on admission, median (Q_25_–Q_75_)	4 (3–7)	4 (3–7)	10 (5–16)	−7.610	<.001
Thrombolytic therapy, *n* (%)	33 (4.3%)	25 (3.7%)	8 (8.7%)	4.822	.027
Dysphagia, *n* (%)	83 (10.8%)	36 (5.3%)	47 (51.1%)	175.339	<.001
Use of nasogastric tube, *n* (%)	70 (9.1%)	30 (4.5%)	40 (43.5%)	148.492	<.001
Baseline SBP (mmHg), median (Q_25_–Q_75_)	150 (140–170)	150 (140–169)	155 (139–170)	−0.909	.363
Baseline DBP (mmHg), median (Q_25_–Q_75_)	89 (80–95)	90 (80–95)	85 (80–92)	−1.340	.180
**Stroke subtype, *n* (%)**					
Atherosclerosis	308 (40.2%)	263 (39.0%)	45 (48.9%)		<.001
Cardioembolism	133 (17.4%)	102 (15.1%)	31 (33.7%)		
Small vessel occlusion	315 (41.1%)	299 (44.4%)	16 (17.4%)		
Other determined	5 (0.7%)	5 (0.7%)	0		
Undetermined	5 (0.7%)	5(0.7%)	0		
**Medical history**					
Previous stroke, *n* (%)	179 (23.4%)	156 (23.1%)	23 (25%)	0.155	.693
Hypertension, *n* (%)	538 (70.2%)	470 (69.7%)	68 (73.9%)	0.677	.411
Diabetes, *n* (%)	183 (23.9%)	158 (23.4%)	25 (27.2%)	0.620	.431
Coronary heart disease, *n* (%)	38 (5.0%)	33 (4.9%)	5 (5.4%)	0.050	.823
Atrial fibrillation, *n* (%)	106 (13.8%)	79 (11.7%)	27 (29.3%)	21.095	<.001
Smoking, *n* (%)	205 (26.8%)	192 (28.5%)	13 (14.1%)	8.512	.004
Drinking, *n* (%)	160 (20.9%)	149 (22.1%)	11 (12.0%)	5.047	.025
A^2^DS^2^ score, median (Q_25_–Q_75_)	3 (1–4)	2 (1–4)	5 (3–7)	− 8.832	<.001
**Laboratory data**					
WBC (×10^9^/L), median (Q_25_–Q_75_)	6.40 (5.23–8.04)	6.30 (5.20–7.86)	7.32 (6.14–10.38)	−4.191	<.001
Neutrophil (×10^9^/L), median (Q_25_–Q_75_)	4.27 (3.25–5.70)	4.20 (3.20–5.47)	5.85 (3.97–8.57)	−5.089	<.001
Lymphocyte (×10^9^/L), median (Q_25_–Q_75_)	1.40 (1.00–1.82)	1.44 (1.05–1.89)	1.00 (0.68–1.50)	−5.889	<.001
Monocyte (×10^9^/L), median (Q_25_–Q_75_)	0.39 (0.30–0.50)	0.39 (0.30–0.50)	0.40 (0.31–0.51)	−1.858	.063
Platelet (×10^9^/L), median (Q_25_–Q_75_)	178 (143–217)	180 (143–219)	170 (138–210)	−1.482	.138
Hemoglobin concentration (g/L), median (Q_25_–Q_75_)	135 (124–146)	136 (125–146)	125 (112–140)	−4.762	<.001
Hs‐CRP (mg/L), median (Q_25_–Q_75_)	1.50 (0.50–3.60)	1.20 (0.50–2.93)	4.90 (2.63–8.40)	−9.281	<.001
ALB (g/L), median (Q_25_–Q_75_)	38.7 (36.8–40.7)	39.0 (37.1–41.0)	36.9 (32.9–38.4)	−7.541	<.001
CAR (10^−5^), median (Q_25_–Q_75_)	3.7 (1.3–9.3)	2.9 (1.2–7.6)	13.6 (7.2–25.1)	−9.640	<.001

Abbreviations: ALB, albumin; A^2^DS2, age, atrial fibrillation, dysphagia, sex, stroke severity; CAR, C‐reactive protein‐albumin ratio; Hs‐CRP, hypersensitive C‐reactive protein; NIHSS, National Institutes of Health Stroke Scale.

However, the white blood cell (WBC) count, neutrophil count, and Hs‐CRP level were significantly higher in the SAP group than in the non‐SAP group (Table 1). In addition, the lymphocyte count, hemoglobin concentration, and ALB were significantly lower in the SAP group than in the non‐SAP group. The CAR value was also significantly higher in the SAP group than in the non‐SAP group. The other hematological indices were not significantly different between the two groups (*p* > .05).

### 3.2 Relationship between CAR and SAP risk

**FIGURE 2:**
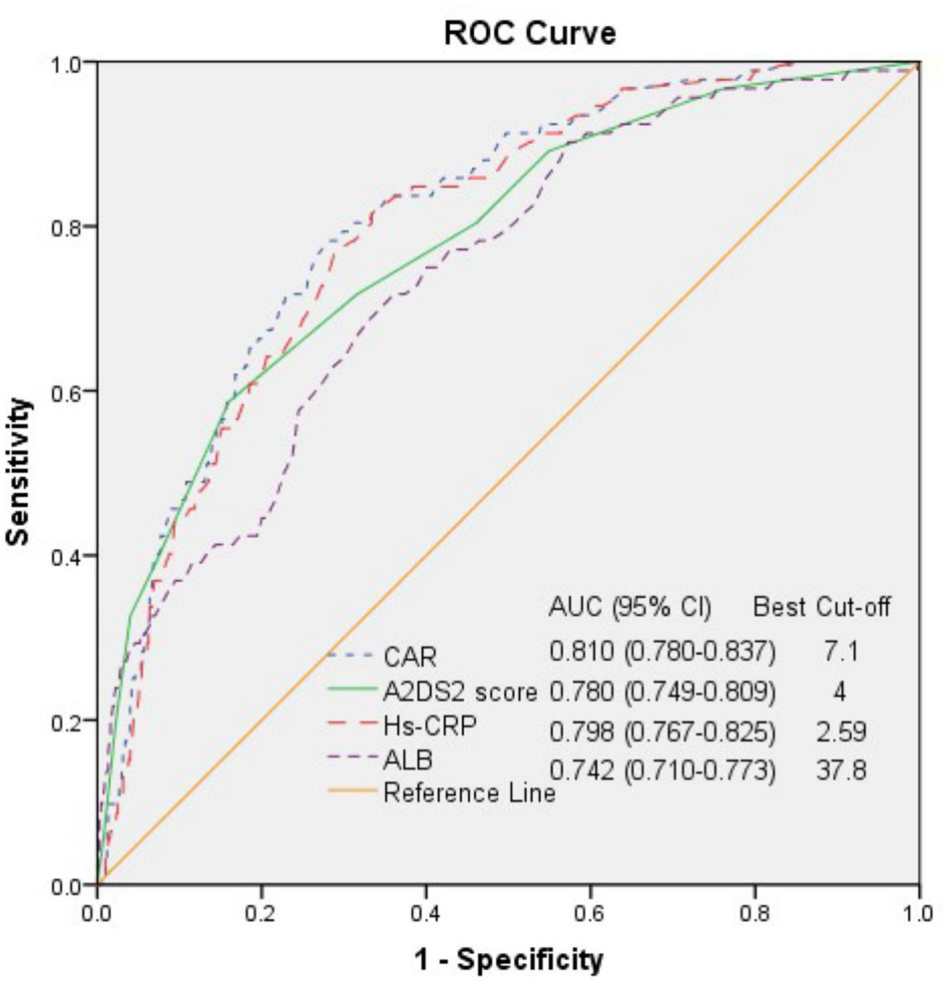
Percentage of different C‐reactive protein‐albumin ratio (CAR) quartile in patients with stroke‐associated pneumonia (SAP) and non‐SAP (*p* < .001)

The CAR level was significantly higher in the SAP group than in the non‐SAP group (13.6 [7.2–25.1] vs. 2.9 [1.2–7.6], *P *< .001) (Table 1). The patients were divided into four groups based on the quartiles of admission CAR level (Q1 <1.3, Q2 1.3–3.7, Q3 3.7–9.3, Q4 ≥9.3) for statistical analysis (the detailed grouping information is shown in Figure 2). Only 2.2% of the SAP patients were in Q1, and over 50% were in Q4 (2.2 vs. 12.0 vs. 20.7 and 65.1%, respectively; *p <*.001). However, the non‐SAP group had a different data distribution. The Q1 group had the highest proportion of non‐SAP patients (28.2 vs. 26.7 vs. 25.7 and 19.4%, respectively; *p <*.001).

The results from comparing the baseline data showed that over 10 risk factors had *p* < .05. However, the sample size was only 92 SAP samples. As the A^2^DS^2^ score contains age, AF, dysphagia, sex, and NIHSS score, we used the A^2^DS^2^ score for multivariate analysis. Similarly, Hs‐CRP and ALB were not included in the multivariate analysis since the CAR is the ratio of Hs‐CRP to ALB. The study aimed to investigate the association between the CAR and SAP development during hospitalization. Finally, in addition to the CAR quartiles, the A^2^DS^2^ score, first symptom to admission time, thrombolytic therapy, use of nasogastric tube, smoking history, drinking history, WBC count, neutrophil count, lymphocyte count, and hemoglobin concentration were used in the multivariate logistic regression analysis. There was no multicollinearity between the 11 risk factors.

The univariate unadjusted and multivariate adjusted odds ratios for the association between the CAR quartiles and SAP are shown in Table 2. The SAP incidence in the unadjusted model was significantly higher in the Q2, Q3, and Q4 groups (*p <*.05) (OR values; 5.81, 10.43, and 43.51, respectively) than in the Q1 group (*p‐*trend < .001). The SAP incidence in the adjusted model was higher in the Q2 group (aOR, 4.10) than in the Q1 group. However, the difference was not statistically significant (*p >*.05). The Q3 and Q4 groups had ORs of 5.21 and 17.72, respectively, which were also significant (*p <*.05, *p‐*trend < .001).

**TABLE 2:** Univariate analysis and multivariate analysis for the association between C‐reactive protein‐albumin ratio (CAR) quartiles and stroke‐associated pneumonia (SAP) risk

	SAP, *n* (%)	Unadjusted OR (95% CI)		*p*	*p‐*Trend	Adjusted OR (95% CI)	*p*	*p‐*Trend
Q1 <1.3 (*n* = 192)	2 (2.2%)		1 (reference)		<.001	1 (reference)		<.001
Q2 1.3–3.7 (*n* = 191)	11 (12.0%)		5.81 (1.27–26.55)	.023		4.10 (0.86–19.54)	.077	
Q3 3.7–9.3 (*n* = 192)	19 (20.7%)		10.43 (2.40–45.45)	.002		5.21 (1.14–23.89)	.033	
Q4 ≥ 9.3 (*n* = 191)	60 (65.1%)		43.51 (10.45–181.15)	<.001		17.72 (4.02–77.99)	<.001	

*Note*: Adjusted for A^2^DS^2^ score, first symptom to admission time, thrombolytic therapy, use of nasogastric tube, smoking history, drinking history, WBC count, neutrophil count, lymphocyte count, and hemoglobin concentration.

Abbreviation: OR, odds ratio.

**FIGURE 3:**
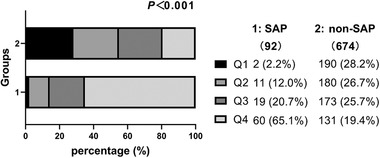
Receiver operating characteristic (ROC) curves for the hypersensitive C‐reactive protein (Hs‐CRP), albumin (ALB), C‐reactive protein‐albumin ratio (CAR), and age, atrial fibrillation, dysphagia, sex, stroke severity (A^2^DS^2^) score in predicting stroke‐associated pneumonia (SAP)

The ROC curve analysis showed that the SAP group had an optimal A^2^DS^2^ cutoff score of 4 (95% CI = 0.749–0.809), an AUC (Area Under Curve) of 0.780, 58.70% sensitivity, and 83.98% specificity (Figure 3). In addition, the AUCs of CAR, Hs‐CRP, and ALB were 0.810 (95% CI: 0.780−0.837), 0.798 (95% CI: 0.767−0.825), and 0.742 (95% CI: 0.710−0.773), with the best cutoff points of 7.1, 2.59, and 37.8, respectively. The AUC comparison is shown in Table 3. The AUC for the CAR was the highest. However, the AUCs were not significantly different between the CAR and A^2^DS^2^ score (*p* > .05) and were significantly different between the CAR and Hs‐CRP or ALB (*p <*.05). Both the A^2^DS^2^ score and the CAR on admission could predict SAP occurrence in hospitalized patients with AIS within 72 h after onset with similar predictive efficacy since their AUROCs were over 0.75 (0.780 vs. 0.810) (*p <*.05). However, the CAR has a more balanced sensitivity and specificity than the A^2^DS^2^ score (77.17% sensitivity, 73.44% specificity vs. 58.70% sensitivity, 83.98% specificity).

**TABLE 3:** Comparison of AUC areas for stroke‐associated pneumonia (SAP) prediction

	AUC area	Difference between areas	*Z*	*p*
CAR vs. A^2^DS^2^ score	0.810 vs. 0.780	0.030	0.867	.386
CAR vs. Hs‐CRP	0.810 vs. 0.798	0.012	5.206	<.001
CAR vs. ALB	0.810 vs. 0.742	0.068	2.136	.032

*Note*: The DeLong test was used to compare AUCs.

Abbreviations: ALB, albumin; A^2^DS^2^, age, atrial fibrillation, dysphagia, sex, stroke severity; CAR, C‐reactive protein‐albumin ratio; Hs‐CRP, hypersensitive C‐reactive protein.

### 3.3 Short‐term clinical outcomes in patients with SAP and non‐SAP

There were no significant differences in the length of hospital stay or the incidence of TIA or AIS within 3 months after discharge between the two groups (*p* > .05) (Table 4). However, the NIHSS and mRS scores at discharge and the mRS score 3 months after discharge were significantly different between the two groups (*p <*.001). At the 3‐month follow‐up after discharge, 200 (26.1%) participants experienced poor functional outcomes (mRS 3–6), and 105 (13.7%) patients died from all causes. The incidence of poor clinical outcomes (mRS 3–6) was significantly higher in the SAP group (69.6% vs. 20.2%, *p <*.001) with a high mortality rate compared to the non‐SAP group (53.3% vs. 8.3%, *p <*.001).

**TABLE 4:** Short‐term clinical outcomes in patients with stroke‐associated pneumonia (SAP) and non‐SAP

	Total (*n* = 776)	non‐SAP (*n* = 674)	SAP (*n* = 92)	*Z/χ* ^2^	*p*
Hospitalization duration (days), median (Q_25_–Q_75_)	11 (9–14)	11 (9–14)	12 (7–17)	−1.105	.269
Discharge NIHSS score, median (Q_25_–Q_75_)	3 (2–6)	3 (2–5)	10 (4–18)	−8.691	<.001
Discharge mRS score, median (Q_25_–Q_75_)	2 (1–3)	1 (1–3)	4 (2–5)	−8.694	<.001
mRS score at 3 months, median (Q_25_–Q_75_)	1 (0–3)	1 (0–2)	6 (2–6)	−9.208	<.001
Ischemic stroke/TIA in 3 months, *n* (%)	14 (1.8%)	13 (1.9%)	1 (1.1%)	0.319	.572
Mortality at 3 months, *n* (%)	105 (13.7%)	56 (8.3%)	49 (53.3%)	138.289	<.001
Poor clinical outcome (mRS 3–6) at 3 months, *n* (%)	200 (26.1%)	136 (20.2%)	64 (69.6%)	102.343	<.001

Abbreviations: mRS, Modified Rankin Scale; NIHSS, National Institutes of Health Stroke Scale.

### 3.4 Relationship between CAR and 3‐month poor clinical outcomes

The univariate unadjusted and multivariate adjusted odds ratios for the association between the CAR quartiles and 3‐month poor clinical outcomes, including death and major disability (mRS 3–6) and 3‐month all‐cause mortality (mRS =6), are shown in Table 5. The univariate regression analysis showed that the high quartiles of CAR were significantly associated with poor clinical outcomes (unadjusted OR for Q3 vs. Q1, 2.03; Q4 vs. Q1, 3.72; *p*‐trend < .001). After adjusting for SAP during the hospital stay, the association was only significantly higher in Q4 (aOR 1.97, 95% CI 1.15–3.37, *p‐*trend = .014).

**TABLE 5:** Univariate analysis and multivariate analysis for the association between C‐reactive protein‐albumin ratio (CAR) Quartiles and 3‐month poor clinical outcomes

	3‐month poor clinical outcomes, *n* (%)	Unadjusted OR (95% CI)	*p*	*p‐*Trend	Adjusted OR (95% CI)	*p*	*p‐*Trend
	**Death or major disability (mRS 3−6)**						
Q1 <1.3 (*n* = 192)	29 (15.1%)	1 (reference)		<.001	1 (reference)		.014
Q2 1.3–3.7 (*n* = 191)	44 (23.0%)	1.68 (1.00–2.83)	.05		1.49 (0.88–2.54)	.137	
Q3 3.7–9.3 (*n* = 192)	51 (26.4%)	2.03 (1.22–3.38)	.006		1.65 (0.98–2.78)	.062	
Q4 ≥9.3 (*n* = 191)	76 (40%)	3.72 (2.28–6.06)	<.001		1.97 (1.15–3.37)	.013	
	**Death (mRS = 6)**						
Q1 <1.3 (*n* = 192)	8 (4.2%)	1 (reference)		<.001	1 (reference)		.001
Q2 1.3–3.7 (*n* = 191)	19 (9.9%)	2.54 (1.08–5.96)	.032		2.10 (0.88–5.01)	.094	
Q3 3.7–9.3 (*n* = 192)	26 (13.5%)	3.60 (1.59–8.18)	.002		2.62 (1.13–6.10)	.025	
Q4 ≥9.3 (*n* = 191)	52 (27.2%)	8.60 (4.00–18.70)	<.001		3.76 (1.64–8.64)	.002	

*Note*: Adjusted for SAP during hospitalization.

Abbreviations: CI, confidence interval; OR, odds ratio.

The CAR quartiles were also significantly associated with 3‐month all‐cause mortality in the unadjusted model (unadjusted OR for Q2 vs. Q1, 2.54; Q3 vs. Q1, 3.60; Q4 vs. Q1, 8.60; *p*‐trend < .001). Furthermore, the CAR was still significantly associated with 3‐month all‐cause mortality in the adjusted model, and the patients in a higher CAR quartile had a higher risk of death in the first 3 months after AIS (adjusted OR for Q3 vs. Q1, 2.62; Q4 vs. Q1, 3.76; *p*‐trend = .001).

## 4 DISCUSSION

Studies have shown that infection is not only a risk factor for stroke but also a determinant of the prognosis after stroke (Elkind et al., 2020). Among them, SAP has a greater impact on clinical outcomes, as it is more common and difficult to treat (Hilker et al., 2003). The incidence of SAP varies from 7% to 38% in different studies, and a recent study of 4,224,924 patients with AIS found that 149,169 (3.53%) of the patients had SAP during hospitalization (Hannawi et al., 2013; Nam et al., 2018). The differences in the incidence of SAP may be related to a variety of factors, such as the study sample, SAP diagnostic criteria, and hospitalization environment, and the rate was higher among patients admitted to ICUs due to complex factors (Hannawi et al., 2013; Patel et al., 2020). The incidence of SAP was 11.9% in our study, as this was a single‐center study with a small sample size taken from the general ward of the emergency and neurology department, and mild stroke was more common in this study (NIHSS score, 4). Patients admitted to the ICU with severe, unstable vital signs were excluded, but they could have a higher SAP incidence due to coma, dysphagia, use of ventilators, and many other reasons (Hannawi et al., 2013).

There is no doubt that SAP is associated with aspiration due to dysphagia. Previous studies have shown that patients with dysphagia after a stroke have a threefold increased risk of developing pneumonia and up to an 11‐fold increased risk of aspiration pneumonia compared with nondysphagia patients (Martino et al., 2005), and early dysphagia screening can reduce SAP occurrence and improve the prognosis of stroke patients (Al‐Khaled et al., 2016). In our study, over half (51.1%) of the SAP patients had dysphagia, although most of these patients had indwelling gastric tubes. Other researchers have also reported that the respiratory infection rate was significantly higher in tube‐fed stroke patients than in orally fed patients, possibly because they were older, had more severe stroke and dysphagia, and stayed in bed longer, which might be due to the late insertion of the gastric tube (Brogan et al., 2014). However, it is unknown whether the placement of a nasogastric tube increases the pneumonia risk by promoting colonization of the oropharynx with pathogenic bacteria since bacteria are the most common pathogens that cause aspiration pneumonia (Chang et al., 2013), and the SAP incidence is higher in patients who have more missing teeth and poor oral hygiene (Wagner et al., 2016). Furthermore, feeding tubes do not prevent gastroesophageal reflux aspiration (Arnold et al., 2016) since the nasogastric tube that has been inserted can cause dysfunction of the esophageal sphincters and desensitization of the pharyngoglottal adduction reflex (Gomes et al., 2003).

Risk factors such as older age, male sex, chronic obstructive pulmonary disease, smoking, stroke‐induced immunodepression syndrome (SIDS), decreased monocytic human leukocyte antigen‐DR isotype (HLA‐DR), and medications, such as acid‐suppressive medication, have also been proven to be associated with SAP (Eltringham et al., 2019; Gong et al., 2016). In this study, the SAP patients were older, had higher NIHSS scores on admission, and had shorter waiting times at home. Moreover, the thrombolytic treatment rate was higher in the SAP group than in the non‐SAP group, possibly due to severe symptoms and more timely admission. AF, smoking, and drinking were also associated with SAP development in patients with AIS. AF is associated with cardioembolic stroke with cortical infarctions and a higher stroke severity, but it can still affect SAP occurrence even after adjusting the NIHSS score (Hoffmann et al., 2012). A meta‐analysis showed that alcohol consumption increases the risk of pneumonia (Simou et al., 2018). Smoking is a scoreable risk factor in the AIS‐APS scoring system because it may affect the ability of respiratory barrier function to protect against infection (Ji et al., 2013). However, in our study, the SAP group had lower smoking and alcohol consumption rates, and we did not find a specific reason for these different findings.

Early detection and treatment of SAP results in better outcomes, and there are consensus guidelines for the use of antibiotics in SAP treatment (Kishore et al., 2019). Nonetheless, several randomized trials that have evaluated the use of preventive antibiotics have failed to show that the clinical outcomes improved with the use of preventive antibiotics in acute ischemic stroke patients (Kalra et al., 2015). While pneumonia risk scores, such as the A^2^DS^2^ score, can clinically predict SAP occurrence, conditions, such as dysphagia, are often not promptly and accurately evaluated; thus, the delay in identifying high‐risk SAP patients influences the prevention and treatment of SAP (Bray et al., 2017). At present, we need to find an indicator that can predict SAP early and accurately.

In addition to dysphagia, stroke‐induced immunodepression syndrome (SIDS) also causes SAP (Liu et al., 2018). Studies have shown that SIDS leads to a rapid and sustained depression of cellular immune functions (Hoffmann et al., 2017). Deactivation of monocytes and Th1 cells, Th1‐mediated lymphopenia, and increased apoptosis of immune cells in the lymph nodes spleen and thymus are all involved (Liesz et al., 2009). In addition to reducing inflammatory reactions to protect brain tissues, SIDS weakens pathogen resistance in the human body, causing infection (Liu et al., 2018). Therefore, immunologically relevant hematologic and cytologic markers have been of interest for the early prediction of SAP occurrence. Biomarkers that can reflect the immune response and systemic inflammation, such as CRP, IL‐6, NLR, MLR, MHR, natural killer cell number, and T‐lymphocyte subsets, have been proven to be associated with SAP occurrence. In addition, MRPROADM, SUPAR, SAA, and other infectious markers are also predictive indicators of SAP (Hotter et al., 2020).

CRP is one of the most readily available and widely used markers of inflammation, and an increase in CRP is associated with the development of SAP (Kalra et al., 2019). Previous studies have found that Hs‐CRP is an independent prognostic marker of functional outcome and death in Chinese patients with AIS (Tu et al., 2013). Hypoalbuminemia is associated with an increased risk of infections, especially pneumonia. The serum ALB level is an independent nosocomial pneumonia predictor in stroke patients. Serum albumin levels are also associated with mortality in aspiration pneumonia patients (Kim et al., 2018). In this study, a composite index, including immune and nutritional status, was identified for SAP occurrence prediction. The hematological marker differences, especially inflammatory markers and nutritional markers, between the two groups were also assessed. The leukocyte count, neutrophil count, lymphocyte count, hemoglobin concentration, ALB, and Hs‐CRP were significantly different between the two groups. In this study, high Hs‐CRP levels and low ALB levels were all SAP risk factors, and thus CAR can predict SAP occurrence in patients with AIS. A higher baseline CAR level was associated with SAP in patients with AIS, with 65.1% of the SAP patients in Q4. In addition, the CAR was an independent SAP predictor in the adjusted model. However, the A^2^DS^2^ score with the most risk factors was significantly different between the two groups. The ROC curves showed that both the CAR and A^2^DS^2^ scores could predict SAP occurrence in patients with AIS with an AUC area over 0.75, showing no significant difference. However, the CAR had a more balanced sensitivity and specificity. In addition, the CAR is easily accessible and simple to use during a hospital stay. Therefore, the CAR is more efficient.

Unlike other reports (Nam et al., 2018; Teh et al., 2018), the length of stay in the SAP group was not prolonged, possibly because our study did not include patients hospitalized in the ICU, who tend to have a longer hospitalization duration. A study found no association between stroke‑associated infection and poor functional outcome at discharge (Vargas et al., 2006). In this study, the NIHSS and mRS scores in the SAP group were higher at discharge, but this did not imply that they are related to SAP because the NIHSS score was also higher at admission. In addition, other complications, such as stroke progression, hemorrhagic transformation, and secondary epileptic seizure, can also affect the disease severity at discharge. Stroke‑associated infection independently predicts 3‐month poor functional outcome and mortality (Suda et al., 2018), and the SAP group had a higher mortality rate and a higher rate of poor clinical outcomes at the 3‐month follow‐up in our study. High CAR levels were related to poor functional prognosis and increased mortality at 3 months after discharge in our study (*p*‐trend < .05), and the results were still statistically significant after adjusting for SAP, especially in the Q4 group. However, this does not indicate that the CAR at admission predicts the risk of a poor clinical outcome at 3 months because there are many other confounding factors. Age, sex, physical state, severity of cerebral infarction symptoms, types, and severity of complications during hospitalization, family care after discharge, rehabilitation treatment, and some other factors may also be associated with the clinical outcome of the patients in the first 3 months after AIS. Therefore, more information is needed during and after hospitalization to clarify the impact of SAP on patients with AIS.

Similar to previous studies, our study also suggests that inflammatory markers are associated with SAP occurrence and short‐term prognosis in patients with AIS. Unlike previous studies, we proposed for the first time to use the CAR, a composite index containing the inflammatory indicator CRP and the nutritional indicator ALB, as a predictor. Through this study, we can cautiously believe that the CAR level at admission is related to SAP occurrence in patients with AIS during hospitalization and the 3‐month prognosis of these patients.

However, there are still several limitations in our study. First, this was a single‐center and retrospective study, and it was difficult to establish causality between the CAR and SAP. Therefore, multicenter, prospective studies are needed in the future. Second, the Hs‐CRP and ALB levels were measured only at baseline, and we did not measure the levels of Hs‐CRP and ALB during hospitalization and follow‐up, which may also be associated with SAP and clinical outcomes. Third, the latest inflammatory factors, such as IL‐6, IL‐1, tumor necrosis factor alpha (TNF‐α), and the more sensitive infection markers, including procalcitonin, were not included in this study. More research is needed to explain the relationship between inflammation and SAP. Fourth, high CAR levels were associated with poor clinical outcomes at 3 months of discharge, regardless of whether SAP was adjusted for. More information, such as complications during hospitalization and after discharge, including the care and rehabilitation treatment, complications, and hospital readmission after discharge, should be collected to determine the relationship between the CAR and prognosis of patients with AIS.

## 5 CONCLUSION

In conclusion, patients with AIS with a higher level of CAR at admission were more likely to develop SAP during hospitalization, had poorer clinical outcomes in the short term, and had higher rates of mortality and severe disability at 3 months. Measuring the CAR at admission and the use of dynamic retesting of the CAR during hospitalization may help in the timely selection of high‐risk patients who need intervention, and this may reduce the occurrence of SAP and improve the treatment outcome of patients with AIS. The CAR can also be retested when the patient is discharged from the hospital, and the patient's inflammation and malnutrition can also be treated after discharge to obtain a better prognosis. Although the CAR was found to be associated with 3‐month mortality and 3‐month unfavorable clinical outcomes after admission, more data are needed to explore their association.

## CONFLICT OF INTERST

The authors declare no conflict of interest.

## AUTHOR CONTRIBUTIONS

Lingling Huang and Rong Zhang are co‐first authors. Rong Zhang and Yaming Sun provided funding and designed the study. Ge Xu, Juan Lu, and Yadong Wang collected the data. Lingling Huang, Fengdan Long, and Jiahui Ji were involved in data cleaning, follow‐up, and verification. Yaming Sun revised the article. All authors have read and approved the final manuscript.

### PEER REVIEW

The peer review history for this article is available at https://publons.com/publon/10.1002/brb3.2675.

## Data Availability

Lingling, Huang (2022), CAR and SAP, Dryad, Dataset, https://doi.org/10.5061/dryad.6wwpzgn23
